# Epigenetic modification mechanisms involved in keloid: current status and prospect

**DOI:** 10.1186/s13148-020-00981-8

**Published:** 2020-11-26

**Authors:** Wenchang Lv, Yuping Ren, Kai Hou, Weijie Hu, Yi Yi, Mingchen Xiong, Min Wu, Yiping Wu, Qi Zhang

**Affiliations:** Department of Plastic and Aesthetic Surgery, NO 1095 Jiefang Avenue, Tongji Hospital, Tongji Medical College, Huazhong University of Science and Technology (HUST), Wuhan, 430000 Hubei China

**Keywords:** Keloid, Epigenetic modification, DNA methylation, Histone modification, ncRNAs

## Abstract

Keloid, a common dermal fibroproliferative disorder, is benign skin tumors characterized by the aggressive fibroblasts proliferation and excessive accumulation of extracellular matrix. However, common therapeutic approaches of keloid have limited effectiveness, emphasizing the momentousness of developing innovative mechanisms and therapeutic strategies. Epigenetics, representing the potential link of complex interactions between genetics and external risk factors, is currently under intense scrutiny. Accumulating evidence has demonstrated that multiple diverse and reversible epigenetic modifications, represented by DNA methylation, histone modification, and non-coding RNAs (ncRNAs), play a critical role in gene regulation and downstream fibroblastic function in keloid. Importantly, abnormal epigenetic modification manipulates multiple behaviors of keloid-derived fibroblasts, which served as the main cellular components in keloid skin tissue, including proliferation, migration, apoptosis, and differentiation. Here, we have reviewed and summarized the present available clinical and experimental studies to deeply investigate the expression profiles and clarify the mechanisms of epigenetic modification in the progression of keloid, mainly including DNA methylation, histone modification, and ncRNAs (miRNA, lncRNA, and circRNA). Besides, we also provide the challenges and future perspectives associated with epigenetics modification in keloid. Deciphering the complicated epigenetic modification in keloid is hopeful to bring novel insights into the pathogenesis etiology and diagnostic/therapeutic targets in keloid, laying a foundation for optimal keloid ending.

## Introduction

Keloid is a common dermal fibroproliferative disorder characterized by the aggressive proliferation of fibroblasts and the excessive accumulation of ECM, like collagen [1]. Keloid is more likely to occur in areas of the body where the skin is tight and repeatedly stretched, namely the front chest, shoulder deltoid, abdomen, and ear. The characteristic growth pattern of keloid is closely associated with the magnitude of skin tension and the direction of mechanical strain. On account of continuing to grow and expanding without spontaneous resolution, patients are usually accompanied by itching and pain, especially keloid contractures located near the joints may lead to serious dysfunction, seriously affecting the patient’s physiological and psychological health. Presently, although several commonly therapeutic approaches have been applied for keloid prevention and treatment, principally including surgical excision combined with local radiotherapy, steroid injections, and compression therapy, they have been proved to be largely ineffective and easy relapse [2].

Epigenetics, the study of the alteration of gene expression without changing the nucleotide sequence, is becoming a very important mechanism accounting for the complexity in many diseases, such as cancer, diabetes, and fibrosis [3]. Known epigenetic modification is mainly composed of three processes, namely DNA methylation, histone modification (including methylation, acetylation, phosphorylation, ubiquitination, and SUMOylation), and ncRNAs-based mechanisms (including microRNAs-miRNAs, long non-coding RNAs-lncRNAs, and circular RNAs-circRNAs) [4]. Keloid formation may be caused by multiple systemic, environmental, and genetic factors that collectively or individually stimulate wound healing and subsequent persistent inflammation. Epigenetics is supposed to represent the potential link of complex interactions between genetics and external risk factors.

Epigenetic modification including DNA methylations, histone modifications, and ncRNAs regulations, are emerging intriguing research fields of illuminating the molecular pathogenesis of keloid investigation. More and more evidence indicates that epigenetic changes are crucial in the initial and sustained activation of fibroblasts in keloid. It is believed that the epigenetic dysregulation will lead to an imbalance in the process of scar tissue repair and regeneration. The epigenetic mechanism in other diseases is relatively thorough, but the pathogenesis of scars is still not very clear.

In this review, we generalize and summarize the present available clinical and experimental studies that investigating the regulators on epigenetic modification and underlying mechanisms in the initiation and progression of keloid, mainly including DNA methylation, histone modification, and ncRNAs represented by miRNAs, lncRNAs, and circRNAs. Strategically, the detection methods of high-throughput detection strategies and biological enrichment discovery are principally employed to screen and investigate the epigenetic modification in keloid, following by experimental verification. Deciphering the complicated epigenetic modification in keloid is hopeful to bring novel insights into the molecular mechanism of keloid pathogenesis and lay the foundation for the effective and precise diagnosis or treatment of keloid.

## Epigenetic mechanisms with histopathology

Keloid disease is a benign disease with the abnormal fibroproliferative property. The most discerning characteristics of keloids on the histopathology are a thickened, flattened epidermis with the haphazard, thick, hyalinized collagen bundles located in the dermis, accompanied with a vast number of fibroblasts and myofibroblast. Compared to healthy skin, keloid tissues show an increased type I/III collagen ratio, decreased level of fibrillin-1 and decorin, increased expression of fibronectin, versican, elastin, and tenascin in the reticular dermis and hyaluronan and osteopontin in the epidermis [5]. The imbalance between the inflammation, proliferation, and remodeling stages is believed to be responsible for the differences in histological characteristics related to keloid and other skin fibrosis.

Firstly, keloid-derived fibroblasts served as the main cellular components in keloid skin tissue play a pivotal role in modulating the synthesis and remodeling of ECM and wound scar healing after burns, trauma, and surgery [6]. It is worth noting that the continuous transformation and invasive growth of fibroblasts into myofibroblasts is beyond the confines of the original wound, reflecting the characteristics of benign skin tumors of keloid [7]. As the imbalance between fibroblast proliferation and apoptosis is the cytological basis for the continuous proliferation of keloid, it highlights the epigenetic contribution to keloids formation by modulating the balance between fibroblast proliferation and apoptosis [8]. Secondly, keloids also show increased immune cell infiltration, with higher quantities of infiltrated macrophages and T-lymphocytes. In addition, immune cells continue to release cytokines and growth factors, which can result in continuous cell proliferation and ECM deposition. Increased synthesis of ECM collagen is thought to be related to the overactivation of keloid fibroblasts (KFs) via the overexpression of inflammatory mediators like TGF-β1. More recently, the role of epigenetic modification especially DNA methylation, histone modification, and ncRNAs, in the etiology and fibrosis progression of keloid has recently attracted widespread attention. KFs with aberrant expression and activation is regulated by several epigenetic mechanisms, forming the complex dynamic regulatory networks in the epigenetic pathogenesis. Meanwhile, due to the dissatisfactory efficacy to reduce or prevent pathological keloid formation, an enhanced understanding of epigenetics contributing to keloid pathologies will benefit for developing early diagnostic tools and future therapies [9]. For instance, exploiting epigenetic modifiers such as DNA methylation/histone enzyme inhibitors are promising in keloid therapy [10].

## Epigenetic modification expression profile in keloid

### DNA methylation expression profiles in keloid

Epigenetic modification of DNA refers to a chemical modification process in which a methyl group is transferred to a cytosine, most commonly at a CpG (cytosine linked to guanine by a phosphate group, cytosine-phospho-guanine, 5′-C-p-G-3′) site [11]. DNA methylation without base sequence alteration is considered as the most common and highly dynamic epigenetic modification, which is the result of the function of cytosine DNA methyltransferases (DNMTs) [12, 13]. DNMTs contain three members: DNA methyltransferase 1 (DNMT1), DNA methyltransferase 3a (DNMT3a), and DNA methyltransferase 3b (DNMT3b). Among them, DNMT1 is considered to be an enzyme that mainly maintains the methylation status, and vividly metaphorized as methylation maintenance. For DNMT3a and DNMT3b, they primarily play a crucial role in de novo methylation. In the process of DNA methylation, DNMTs first converts cytosine to 5-methylcytosine (5-mC), and then sequentially oxidizes 5mC to 5-hydroxymethylcytosine (5-hmC), 5-formylcytosine (5-fC) and 5-carboxycytosine (5-caC) by the ten-eleven translocation (TET) family [13]. The genome-wide pattern of DNA methylation is generally associated with silencing gene expression since 5-hmC reduces the binding of transcription factors to methylated CpG sites in keloid. For example, the study of Zhang et al. found that keloid tissues with DNA methylation of the CDC2L1 gene promoter region had dramatically lower levels of CDK11p58 protein than healthy normal skin tissues without DNA methylation of the CDC2L1 gene promoter region [14]. Therefore, the aberrant addition (hypermethylation), or removal (hypomethylation) of the methyl group by DNMTs and TET can either decrease or increase the rate of gene expression in keloids. Aberrant DNA methylation is frequently attested in keloid currently detected by methylation sequencing PCR-based methods. Furthermore, methylation profiles may represent early markers for the initiation, development, and progression of keloid pathogenesis. The potential reversibility of the DNA methylation pattern may be beneficial for adjuvant therapy or adjuvant therapy with more conventional therapies (such as surgical resection and steroid injection).

Extensive changes in DNA methylation expression profiles following the loss of skin ability of scarless healing. The functional associations of the methylated genes were primarily concerned with embryonic morphogenesis, synapse functions, and neuron and epithelium development, implying scarless healing depended on DNA methylation regulation [15]. Based on genome-wide scanning of methylated cytosine-phosphoguanine (CpG) sites in keloids, a total of 100,000 differentially methylated CpG sites were identified, of which 20,695 were hypomethylated and 79,305 were hypermethylated. The most functionally enriched methylated genes were strongly involved in the regulation of transcription, DNA-templated, and histone exchange, which shed light on the underlying mechanism of keloid formation and remission [16]. By employing genome-wide differentially methylated gene profiles, Jones et al. recognized 152 significant differentially methylated genes in keloid [17]. These methylated genes were applied to pathway enrichment analysis, and identifying mainly three pathways such as histidine degradation V1, phospholipase C, and P2Y purinergic receptor signaling pathway [17]. Another study by Jones et al. revealed that the differentially methylated CpGs between 6 fresh keloid and 6 normal skin samples corresponded to 152 keloid-specific promoter region genes, of which 96 (63%) are hypomethylated as compared to 56 (37%) hypermethylated highlighting a predominance of hypomethylated genomic landscapes. Interestingly, in this preliminary study, members of short non-coding RNA gene families involved in gene regulation such as miR-199A2, miR-609, and miR-938 were also differentially methylated [18]. Furthermore, Garcia-Rodriguez et al. identified 4 master regulators (pyridoxamine, tributyrin, PRKG2, and PENK) and 19 intermediate regulators in keloid, which involved in cell proliferation, senescence, apoptosis, and tumor suppression, predicting to be closely relevant to the occurrence and progress of keloid [19]. Together, DNA methylation, as an alternative mechanism for gene regulation in keloid pathogenesis, holds the potential to reverse deleterious epigenetic alteration and presents complicated cross talk with other epigenetic modifications, especially miRNAs. Meanwhile, these results provide critical insights into DNA methylation in keloid formation along with the peculiar attentiveness as a potential biomarker for efficacious therapeutic strategies.

### Non-coding RNAs expression profiles in keloid

Only 2% of genomic DNA encodes proteins, while the remaining 98% is transcribed as ncRNAs, which refer to the type of RNAs that are not translated into proteins and regulate gene expressions only at the transcriptional and post-transcriptional level [20, 21]. Certain types of ncRNAs, ubiquitously found in multiple cell types, are considered as housekeeping RNAs, e.g., ribosomal, transfer, small nuclear, small nucleolar RNAs, and ribonuclease P RNAs [22, 23]. In the past, due to sequencing techniques and methodology limitations, studies on RNA epigenetics principally focus on tRNA and rRNA with high abundance and intensive modulation [24, 25]. Currently, empowered by recent advances of sequencing techniques, the intensive research of the transcriptome-wide range has indicated that ncRNAs (miRNAs, lncRNAs, and circRNAs) are crucial in the coordination of KFs function and gene transcription, as well as in the pathogenesis of keloid. According to high-throughput sequencing and gene microarray results, the expression profiles of specific miRNA, lncRNA, and circRNA have altered in keloid tissue and fibroblasts, which may partially promote the etiology of keloid by affecting signaling pathways related to the occurrence and progression of keloid (Table 1). Therefore, the complicated cross talk of ncRNAs, including miRNA, lncRNA, and circRNA, might partially be responsible for the excessive proliferation of fibroblasts and abnormal activation of myofibroblasts for shaping keloid progress.

**Table 1:** The expression profiles of miRNAs, lncRNAs, and circRNAs in keloid

ncRNAs	Sample	Number (cases/controls)	DE ncRNAs (up/down)	Up-regulated ncRNAs verified by qRT-PCR	Down-regulated ncRNAs verified by qRT-PCR	KEGG enrichment analysis	Ref
MiRNAs	Skin tissue	3/3	40 (27/13)	miR-370-3p	miR-204-5p	Regulation of actin cytoskeleton pathway Yersinia infection pathway Proteoglycans in cancer pathway	[37]
	Skin tissue	3/3	12 (6/6)	N/A	miR-194-3p	N/A	[31]
	Skin tissue	3/3	264 (139/125)	miR-199a-5p miR-21-5p miR-214-5p miR-424-5p	miR-205-5p	MAPK signaling pathway HIF-1 signaling pathway Prolactin signaling pathway	[32]
	Skin tissue	8/8	293 (168/125)	N/A	miR-199a-5p	N/A	[30]
	Skin tissue	12/12	32 (23/9)	miR-21 miR-4269 miR-382	miR-203 miR-205 miR-200c	Cell cycle pathway MAPK pathway P53 pathway	[28]
	Skin tissue	5/5	74 (46/28)	N/A	miR-1224-5p	N/A	[76]
	Fibroblast	3/3	9 (6/3)	miR-4328	miR-152 miR-145-5p miR-320c miR-30a-5p	TGF-β pathway MAPK pathway Apoptosis and cell cycle pathway	[29]
	Serum	9/7	37 (17/20)	miR-1225-5p miR195-5p, miR-513-5p	miR-6801-3p miR-4254 miR-412-3p	PI3K-Akt signaling pathway MAPK signaling pathway Ras signaling pathway	[33]
LncRNAs	Skin tissue	3/3	319 (251/68)	lnc-CASP9-3	lnc-GLB1L-1	ErbB pathway Phospholipase D pathway	[37]
	Skin tissue	2/2	2227 (1224/1003)	N/A	N/A	Cancer pathway Metabolic transcriptional misregulation pathway RAS pathway	[41]
	Skin tissue	4/4	30 (16/14)	LOC100271722	HNF1A-AS1	Notch pathway Wnt pathway Hippo pathway	[42]
	Skin tissue	4/4	69 (38/31)	CACNA1G-AS1 HOXA11AS LINC00312	RP11-91I11.1 AP001476.4 RP4-794H19.4 AC004074.3	Wnt pathway	[43]
	Skin tissue and Fibroblast	5/5 and 3/3	3680 (2238/1442) and 5448 (2526/2922)	ENST00000439703 uc003jox.1	N/A	Wnt pathway PI3K-Akt pathway PPAR signaling pathway	[39]
	Skin tissue	3/3	2068 (1290/778)	NONHSAT120157 NONHSAT062994 NONHSAT016933 NR 024360.1 FR39263	NONHSAT053431 FR244962 ENST00000601148 TCONS 00022478 XR 244388.1	Focal adhesion pathway Metabolic pathway ECM-receptor interaction pathway	[40]
	Skin tissue	3/3	2513 (1731/782)	ENST00000522743 NR-038439 uc0021fu.1	ENST00000521141	ECM-receptor interaction pathway Calcium pathway MRNA surveillance pathway	[38]
	Skin tissue	6/6	3469 (2479/990)	ENSG00000251085 ENSG0000 0223749 ENSG0000 0258876 ENSG0000 0237548	ENSG0000 0268262	TNF pathway TGF-β pathway Jak-STAT pathway	[49]
CircRNAs	Fibroblast	3/3	411 (206/205)	circRNA-0008259 circRNA-0005480	circRNA-0002198	Focal adhesion pathway PI3K-Akt pathway Metabolic pathway	[47]
	Skin tissue	3/3	76 (52/24)	N/A	circRNA-0057452, circRNA-0007482, circRNA-0020792, circRNA-0057342 circRNA-0043688	cAMP pathway Cell cycle pathway Cancer pathway	[48]
	Skin tissue	2/2	154 (81/73)	N/A	circRNA-0000994	Focal adhesion pathway Actin cytoskeleton pathway PI3K-Akt pathway	[41]
	Skin tissue	6/6	11 (10/1)	circ17-50190326-50194041 circ17-50189167-50194626 circ17-50189858-50195330 circ17-50189167-50198002	circ11-33286412-33287511 circ2-72718102-72733118	ECM-receptor interaction pathway Gap junction pathway Protein digestion and absorption pathway	[49]

DE ncRNAs were verified to be mainly associated with multiple signaling pathways involved in proliferation, migration, apoptosis, and differentiation through high-throughput sequencing and bioinformatics analysis. Meanwhile, qRT-PCR verification is further methods of confirming the identified dysregulated ncRNAs. *NA* not available, *DE* differentially expressed, *ncRNAs* non-coding RNAs, *miRNAs* microRNAs, *lncRNAs* long non-coding RNAs, *circRNAs* circular RNAs; *qRT-PCR* quantitative reverse transcription-polymerase chain reaction, *KEGG* Kyoto Encyclopedia of Genes and Genomes

#### MiRNA expression profiles in keloid

MiRNAs, which are endogenous ncRNAs with lengths of approximately 18 to 22 nucleotides, are involved in transcriptional and post-transcriptional regulation of gene expression in many skin fibrosis diseases such as scleroderma and keloid [26]. Currently, lots of miRNAs have been identified, annotated in keloid involved in regulating cellular behavior, including aberrant proliferation, myofibroblast activation, autophagy, apoptosis, cell cycle, migration, and collagen production.

Whole-genome expression analysis is a widely used strategy to screen differential gene and miRNA expression in clinical keloid specimens, while microarray analysis and qRT-PCR verification are further methods of confirming the identified dysregulated miRNA [27]. For example, Liu et al. employed miRNA microarray analysis to identify a total of 32 differentially expressed (DE) miRNAs accompanied by 23 miRNAs up-regulated and 9 miRNAs down-regulated in keloid, which were closely associated with TGF-β, MAPK, apoptosis, and cell cycle signaling pathway [28]. The qRT-PCR ulteriorly demonstrated the miRNA abundance including up-regulated miRNA-21, miRNA-4269, and miRNA-382 and down-regulated miRNA-203, miRNA-205, and miRNA-200c. Similarly, in keloid-derived fibroblasts, Li et al. authenticated 9 dramatically DE miRNAs, among of which, miR-152, miR-23b-3p, miR-31-5p, miR-320c, miR-30a-5p, and hsv1-miR-H7 were significantly up-regulated, while miR-4328, miR-145-5p, and miR-143-3p were down-regulated [29]. In Wu et al. study, a total of 17 DE miRNAs including miR-199a-5p, were identified by microarray hybridization [30]. Transfection with a miR-199a-5p mimic could result in lower cell proliferation and longer S and G2/M phases in KFs [30]. By using gene microarray in 3 paired samples, Xu et al. screened miR-194-3p with low expression in keloid [31]. In vitro assay, miR-194-3p inhibited the expression of CDK4 and MMP2 via targeting RUNX2 directly, thereby inhibited the proliferation and migration of fibroblasts [31].

Through applying the same approach, Zhong et al. revealed that 264 miRNAs were significantly altered in keloid [32]. The miRNA-199a-5p, miRNA-21-5p, miRNA-214-5p, miRNA-424-5p, and miRNA-205-5p, were the high-frequency DE miRNAs and were mainly associated with MAPK and HIF-1 signaling pathway by KEGG enrichment analysis [32]. Besides, it was very meaningful that Luan et al. characterized the miRNA expression profiling in the sera from 9 keloid patients and 7 normal controls, identifying the 37 DE miRNA [33]. Among them, miR-1225-5p, miR195-5p, and miR-513-5p were up-regulated, while miR-6801-3p, miR-4254, and miRNA-412-3p were down-regulated, also highlighting serum miRNAs potential of acting as biomarkers for early keloid diagnosis [33]. Thus, the expression profiling of miRNAs in keloid that are potentially implicated in underlying keloid pathogenesis as confirmed by the above methods.

#### LncRNA expression profiles in keloid

LncRNAs are a heterogeneous population of RNA molecules that are 200 nucleotides in length with limited protein-coding potential [34]. Recently, thousands of lncRNAs have been reported to be essential regulatory molecules in modulating cellular physiology and functions through transcriptional activation, transcriptional interference, and intranuclear transport, also thanks to high-throughput sequencing and bioinformatics technologies [35, 36]. Dysregulated expression of lncRNAs is essential during the processes of several human fibrotic diseases, especially keloid.

Through RNA-seq and miRNA-seq, Duan et al. identified keloid-specific RNAs, including 509 lncRNAs, 25 miRNA, and 94 mRNAs in keloid samples, and showed that these lncRNAs mainly enriched in the actin cytoskeleton, yersinia infection, and proteoglycans in cancer by KEGG pathways analysis [37]. Subsequently, based on co-expression analysis and competing endogenous RNA (ceRNA) network construction, it was revealed that EGFR/miR-370-3p/lnc-GLB1L-1 and ITGB5/miR-204/lnc-CASP9-3 might participate in the underlying mechanisms of lncRNAs regulating keloid [37]. By bioinformatic analysis, Liang et al. constructed a coding–non-coding gene co-expression diagram, and found that 1731 lncRNAs constantly up-regulated and lncRNA CACNA1G-AS1 may be crucial for keloid formation [38]. In both keloid tissue and cell assay, Yuan et al. identified that 71 overlapped and DE lncRNAs were involved in the pathogenesis and development of the keloid, offer the stable events in the pathological progress of keloid [39]. Among them, ENST00000439703 and uc003jox.1 were up-regulated as confirmed through qRT-PCR in enlarged samples.

In an earlobe keloid research, 1290 lncRNAs and 1092 mRNAs were up-regulated, and 778 lncRNAs and 419 mRNAs were down-regulated between earlobe keloid and normal tissues [40]. The characterized expression profiles of lncRNA combined with pathway analysis suggested their biological functions and mechanisms were involved in earlobe keloid formation [40]. Wang et al. also analyzed the ncRNA mechanism of earlobe keloid which might differ from chest keloid. In this study, the sequencing results successfully identified 2227 DE lncRNAs, including 1224 up-regulated and 1003 down-regulated in keloid tissue compared with normal skin tissue [41]. However, this study does not fully explain the underlying causes.

Hedgehog (Hh) signaling pathway-related genes, involving many secretory signaling proteins, have important roles in cutaneous fibrosing disorders. In keloid tissue, Huang et al. found differential expression of 33 mRNAs and 30 lncRNAs relating to the Hh pathway, which were verified by gene chip qPCR. Importantly, by binding the upstream target gene of GLI2 and neighboring target gene HNF1A separately, the lncRNA-AC073257.2 and lncRNA-HNF1A-AS1 could both affect cell keloid growth and proliferation [42]. It was also confirmed that Wnt-genes were able to orchestrate proliferation and regeneration in skin wound response and keloid. Sun et al. used a pathway-focused lncRNA microarray to initially showed a total of 116 Wnt-targeted genes and 69 Wnt-related lncRNAs aberrantly expressed in keloid and further confirmed that 4 lncRNAs including CACNA1G-AS1, HOXA11-AS, LINC00312 and RP11-91I11.1 with their six paired Wnt-genes were finally identified as skin-related lncRNA/gene pairs in keloid [43]. As a highly recurrent benign dermal tumor, it was supposed that in keloid, the lncRNA-dependent regulation of Wnt genes might provide a unique and subtle balance between differentiation and proliferation to keep the excessive cell growth consistently benign.

The lncRNAs groups, aberrantly expressed in keloid compared with normal skin tissue, indicated that DE lncRNAs may orchestrate the keloid formation. These sequencing and validation studies provide new insights of lncRNAs into keloid pathology and potential targets for the treatment of keloid.

#### CircRNA expression profiles in keloid

CircRNAs, consisting of covalently closed continuous loops, are a newly identified type of ncRNAs with the ability to encode proteins, regulate gene transcription, and act as a special “sponge” for miRNAs and harbor abundant miRNA binding sites [44]. Unlike the above two linear miRNAs and lncRNAs, due to the 3′ and 5′ ends commonly presented are firmly concatenated, circRNAs exist in multiple species with high abundance, high sequence conservation, and specific expression [45, 46]. Aberrant circRNAs expression plays a crucial regulatory role during the occurrence and development of fibrosis diseases, including hepatic fibrosis, cardiac fibrosis, and keloid.

To investigate the expression profile and role of circRNAs KFs, Zhang et al. performed high-throughput RNA sequencing technology and screened 411 DE circRNAs with 206 upregulation and 205 downregulation. GO and KEGG pathways enrichment analyses revealed that these DE circRNAs were mainly involved in cell apoptosis, focal adhesion, PI3K-Akt and Rap1 pathway, and metabolic signaling pathway [47]. Similarly, Shi et al. performed a circRNA microarray assay to determine circRNA expression in keloid and paired normal skin tissue, finding 52 significantly up-regulated and 24 down-regulated circRNAs in keloid [48]. The further analysis of the circRNA-miRNA network showed that circRNAs could interact with miRNAs, including miRNA-29a, miRNA-23a-5p, and miRNA-1976. The result indicated that these circRNAs were engaged in the pathogenesis of keloid play vital roles in the pathogenesis of keloid [48]. The expression profiles of mRNAs, lncRNAs, and circRNAs are altered with certainty in keloid tissue, which may partly contribute to the etiology of keloid by impacting several signaling pathways relevant to scaring healing. In a high-throughput sequencing research, compared with normal tissue, there were a total of 81 circRNAs were up-regulated, whereas 73 circRNAs down-regulated in keloid [41]. Li et al. also revealed that DE lncRNAs and circRNAs in human hypertrophic scars, by high-throughput sequencing [49]. The results showed that lncRNAs and circRNAs might act as ceRNAs participated in the pathophysiology and development of human HS [49]. The establishment of a co-expression network groundbreakingly proposed an interesting possibility that ncRNAs might possess a bidirectional relationship and participate in cell-to-cell cross talk.

## Epigenetic modification mechanisms in keloid

### Mechanisms of DNA methylation in keloid

DNA methylation is involved in the integration process and various aspects of keloid, including cell proliferation, invasion, myofibroblast activation, collagen deposition, and pigmentation. Russell et al. observed differential methylation of multiple fibrotic genes with significant changes, including HOXA9 and HOXA10 in KFs, which were generally associated with keloid formation [50]. Liu et al. evaluated DNA hydroxymethylation (5-hydroxymethylcytosine, 5-hmC) status of the patients’ keloid and recorded a significant reduction in KFs, in lines with Jones et al. [51]. Simultaneously, they also demonstrated that ten-eleven translocation (TET) induced loss of 5-hmC similar to that seen in naturally occurring keloid, was partially reversed by a recognized epigenetic regulator of ascorbic acid, indicating that epigenetic regulation in keloid might be manipulated at the level of DNA hydroxymethylation [51]. A study by Zhang et al. found the DNA methylation rate of CpG islands in the CDC2L1 gene promoter region was observably higher in KFs, and the lower expression of the CDK11p58 protein was closely related to DNA methylation of the CDC2L1 gene promoter region, dramatically diminishing apoptosis in KFs [14].

Previous research reported that the expression of the Wnt inhibitor SFRP1 expression was obviously decreased in KFs cultured from the keloid nodule. Russell et al. revealed no differential methylation of the SFRP1 gene through preliminary investigating genome-wide ChIP-chip assay of pooled DNA samples in KFs [50]. Subsequently, the appliance of Trichostatin A (TSA) and 5-Aza-2′-deoxycytidine (5-aza-dc) determined that SFRP1 expression in KFs was increased almost 15-fold by TSA but not by 5-aza-dc, speculating that silencing of SFRP1 was not due to hypermethylation but histone modification. However, recent studies by Liu et al. presented that the lost SFRP1 expression due to the hypermethylation of the SFRP1 promoter likewise might play a vital role in the pathological progress of keloid [52]. The treatment of 5-Aza-dc, as an inhibitor of DNA methyltransferase, appeared to conspicuously escalated SFRP1 level and coordinated Wnt/β-catenin activity in KFs. Furthermore, a functional verification experiment ulteriorly authenticated that downgrade DNMT1 instead of DNMT3a or DMNT3b was responsible for the hypermethylation of the SFRP1 promoter and upregulation of SFRP1 expression in KFs [52]. These two findings indicated that DNA methylation and histone acetylation might cooperatively participate in regulating SFRP1 expression, and the mutual relationship seems to be bidirectional with each able to affect the other. Furthermore, in patient populations with dark baseline pigmentation, one common symptom after wound scar healing is dyspigmentation, which contributes to challenges with social reintegration. Carney et al. found that increased expression of POMC was detected in hypertrophic scar compared with normal skin, and existing distinct alteration in methylation of the POMC promoter [53]. However, subsequent experiments regretfully indicated that methylation of POMC did not correlate to pigmentation status.

The above studies provide evidence of DNA methylation for the regulation of the stable pattern of differential gene expression in keloid formation (Table 2). As a pervasive alternative mechanism for gene regulation in keloid pathogenesis, DNA methylation possesses the capacity of becoming novel potential therapies to reverse destructive epigenetic modification.

**Table 2:** Mechanisms and clinical value of DNA methylation in keloid

DNA methylation	Expression	Mechanism	Clinical value	Ref
HOXA9/HOXA10	Hypermethylation	DNA methylation altered wound healing in keloid fibroblasts	Strategies to treat or prevent keloids	[50]
CDC2L1	Hypermethylation	DNA methylation of the CDC2L1 gene promoter resulted in decreased fibroblast apoptosis, thus promoting the development of keloids	The potential therapeutic value in the process of wound healing for preventing keloid development	[14]
SFRP1	Hypermethylation	The SFRP1 promoter methylation significantly promoted the signaling activity of Wnt/β-catenin and the mRNA and protein expression of β-catenin and α-SMA in keloid fibroblasts	A therapeutic target for keloid treatment	[52]
POMC	Hypermethylation	POMC gene promoter methylation would not account for the development of hypopigmentation in keloid	N/A	[53]

DNA methylation without base sequence alteration is considered as the most common and highly dynamic epigenetic modification. The potential reversibility of the DNA methylation pattern may be beneficial for therapeutic choices

### Mechanisms of histone modification in keloid

Histone proteins together with DNA form the basic structure of chromatin. Post-translational histone modifications such as acetylation, methylation, phosphorylation, and ubiquitination, are epigenetic mechanisms that are known to regulate chromatin structure and gene expression by modulating chromatin compaction degree [54]. Histone acetylation is a dynamic process controlled by the counteracting actions of two large families of enzymes-histone acetyltransferases (HATs) and histone deacetylases (HDACs), which has been proved to be the most thoroughly studied histone modification in keloid [55]. On the basis of the subcellular localization, HATs can be divided into type A and type B, of which type A HATs are located in nuclear and type B HATs are located in the cytoplasm. Moreover, HATs can be divided into three main families by homology sequences and functional similarities: Gcn5 related HAT (GNAT) family, MYST family, and P300/CBP family [56]. In humans, there are 18 highly conserved HDAC enzymes. Based on sequence similarities, HDACs are traditionally divided into two families and four classes: histone deacetylase family (Class I—similar to the yeast Rpd3 protein, Class II—similar to the yeast Hda1 protein, and Class IV—similar to both yeast Rpd3 and yeast Hda1 proteins) and Sir2 regulator family (Class III—similar to the yeast Sir2 protein) [57]. HATs and HDACs are able to remove and add acetyl groups to histones, respectively, in this manner emerging as important means of gene regulation. Cumulative evidence has independently delineated that the roles of HDACs in accelerating fibrogenesis and those HDAC inhibitors (HDACIs) effectively prevented fibrosis. Interestingly, existing studies have found that overproduction of histone deacetylases 2 (HDAC2) belonging to Class I proteins was observed in keloids. Fitzgerald et al. assessed in vivo studies on mouse and human skin wounds, and revealed that HDAC2 was significantly overexpressed in both normal and keloid scar tissue [58]. Their ongoing hypothesis was that pharmacological inhibition of HDACs will decrease skin fibrosis [58]. The CUDC-907, a dual inhibitor of PI3K/Akt/mTOR pathway and HDACs, inhibited cell proliferation, migration, invasion, and ECM deposition of in vitro cultured KFs and also suppressed collagen accumulation and disrupted the capillaries of keloid explants ex vivo and in vivo, with the promotion of the acetylation of histone H3 [59].

TSA, belonging to a group of compounds, termed histone deacetylase inhibitors, can effectively inhibit skin fibrosis formation. Diao et al. proposed that TSA caused abrogation of TGF-β1 induced collagen synthesis in KFs, posing the opinion that TSA inhibition of ECM and inducing apoptosis may be an appropriate therapeutic strategy for the management of keloid [60]. Next, they continue to test the ability of TSA to reduce hypertrophic scar formation in a rabbit ear model [61]. TSA could lead to a reduction in hypertrophic scar formation in the rabbit ear model, accompanied by reduced synthesis of type I collagen and fibronectin, suggesting that the anti-fibrogenic effects of TSA administration on hypertrophic scar formation in vivo [61]. Interestingly, Jian et al. revealed that TSA inhibited the proliferation of KFs in a time- and dose-dependent manner, while there were alterations in the expression of numerous miRNA sequences including TSA-regulated miR-30a-5p, in response to TSA. This evidence suggested that histone modifications and ncRNAs were closely interconnected in their regulation and promotion of gene silencing [62]. As the main or auxiliary drug for scar-like treatment, further studies on the in vivo safety and effectiveness of TSA should be more cautious. The epigenetically altered program affects the biochemistry behavior in KFs, involving in an especially altered pattern of histone acetylation.

The profibrotic transcriptional patterns in fibroblasts are regulated by histone modifications via diverse mechanisms (Table 3). Further elucidation of histone modification in keloid may be achieved by determining individual gene and genome-wide patterns of histone modification. Manipulation of the expression of specific epigenetically modified genes such as CUDC-907 and TSA may reverse fibrosis in keloid.

**Table 3:** Mechanisms and clinical value of histone modification in keloid

Histone modification classification	Inhibitor	Target	Mechanism	Clinical value	Ref
Histone acetylation	N/A	N/A	N/A	N/A	
Histone deacetylation	CUDC-907	HDACs	CUDC-907 inhibited cell proliferation, migration, invasion, and ECM deposition in KFs and also disrupted the capillaries of keloid explants ex vivo and in vivo	A candidate drug for systemic keloid therapy	[59]
Histone deacetylation	TSA	HDACs	TSA could also cause abrogation of TGF-β1 induced collagen synthesis and induce apoptosis of proliferating KFs	TSA might increase the sensitivity of keloid to radiotherapy and become primary or adjunctive agents for the management of keloid	[60]
Histone deacetylation	TSA	HDACs	TSA-induced miR-30a-5p regulated apoptosis and proliferation of keloid fibroblasts via targeting BCL2	A potential use for TSA as effective therapeutic strategies for keloids	[62]

HATs and HDACs are able to remove and add acetyl groups to histones, respectively, in this manner emerging as important means of gene regulation. It reveals the precise molecular mechanisms of HDACs inhibitors for clinical keloid treatment. *HATs* histone acetyltransferases, *HDACs* histone deacetylases

### Mechanisms of miRNAs in keloid

Many miRNA expression levels were notably up-regulated in keloid tissue compared with normal skin tissue. Wang et al. demonstrated that the up-regulated miR-152-3p regulated cell proliferation, invasion, and ECM expression including type I collagen, type III collagen, and fibronectin through targeting FOXF1 in KFs, which indicated that miR-152-3p was a novel and promising target for keloid treatment [63]. Zhang et al. reported that miRNA-31 was memorably incremental in keloid tissue and KFs, and participated in regulating the proliferation, apoptosis, and cell cycle of KFs by regulating HIF1AN/VEGF signaling pathway [64]. MiR-181a was also significantly up-regulated in human keloid tissue and KFs [65]. MiR-181a negatively regulated PHLPP2 expression, and enhanced cell proliferation and inhibited apoptosis through activating the AKT pathway, and consequently accelerated cell growth of KFs [65]. However, the functional roles of these miRNAs in vivo require further investigation.

MiR-21 is an important and widely studied miRNA, which has been found to be up-regulated in keloid tissue. TGF-β1 is up-regulated in keloid tissue with the ability to promote the proliferation of skin fibroblasts, collagen formation, and differentiation. The interaction between TGF-β1 and miR-21 in the regulation of FasL protein plays a pivotal role in keloid formation [66]. Furthermore, the up-regulated miR-21 and PTENAKT signaling pathway participated in the TGF-β1 induced KFs proliferation and transdifferentiation, which could be attenuated by miR-21inhibition [67]. Yan et al. reported that transfected miR-21-5p mimic or inhibitor, respectively, increased or decreased the migration, invasion, and sphere-forming abilities of keloid keratinocytes, implicating the participation of PTEN and p-AKT in the miR-21-5p regulation on EMT phenotypes and stemness signatures of keloid keratinocytes, which might be accounted for the invasion and recurrence of keloid [68]. Besides, miR-21 could regulate the KFs apoptosis via targeting FasL, caspase-8, and the mitochondria-mediated apoptotic signaling pathway, suggesting that the possibility ofmiR-21 considered as a therapeutic target for keloid [69]. Meanwhile, another study also revealed that miR-21 enhanced collagen production in keloid through negatively regulating Smad7 [70].

Some specific miRNAs are down-regulated in keloid tissue involved in negatively regulating keloid formation. Downregulation of miR-196a may be one of the mechanisms by which collagens are highly deposited in keloid tissue, as the reporter analysis showed miR-196a upregulation reduced the expression of collagen in KFs through binding to the 3′UTR of COL1A1 and COL3A1 [71]. Li et al. provided evidence to support the target potential of miR-200b for hypertrophic scarring management, attributing to that overexpression of miR-200b postponed the fibrosis progress through regulating the cell proliferation, apoptosis, and TGF-β1/α-SMA signaling [72]. Zhang et al. also emphasized that miR-29a was dramatically decreased in KFs and knockdown with anti-miR-29a miRNA promoted keloid fibrogenesis, thus miR-29a might exert as a novel regulator in the keloid fibrogenesis process [73]. The upregulation of miR-205 was capable of suppressing keloid formation by reducing VEGF expression-mediated PI3K/Akt signaling transduction in KFs, demonstrating that miR-205-5p inhibited the pathogenesis of keloid [74]. In a similar study, the down-regulated miR-141-3p was found in keloid and the elevated miR-141-3p would result in markedly decreased proliferation, migration, and the promotion of apoptosis via targeting GAB1 in KFs [75]. Feng et al. also assured that the overexpression of miR-1224-5p, with low expression level in keloid tissue, inhibited KFs proliferation and promoted apoptosis, suggesting the downregulation of miR-1224-5p involved in the occurrence and development of keloid [76].

In recent studies, descending levels of miR-203 have been verified in both keloid tissue and KFs through qRT-PCR. Shi et al. substantiated that overexpressed miR-203 played a negative regulatory role in proliferation, invasion, and ECM production by repressing EGR1 and FGF2 expression in KFs [77]. In addition, miR-152-5p and miR-637 had been demonstrated to be decreased in KFs. The upregulation of miR-152-5p played an irreplaceable role in putting off the progression of fibrosis in keloid by inhibiting proliferation, migration, and promoting apoptosis through the Erk1/2 and Akt pathways [78]. Interestingly, both miR-637 mimic and silencing Smad3 obtained consistent results in KFs, thereby confirmed that Smad3 was the direct target of miR-152-5p and miR-637 [79]. MiR-188-5p, known as a tumor-suppressive factor, was observably down-regulated in keloid and hypertrophic scars tissue. The increased expression of miR-188-5p transfected with miR-188-5p mimic exerted an inhibitory effect on the regulation of proliferation, migration, and invasion in KFs by restraining PI3K/Akt/MMP-2/9 signaling pathway [80]. Liu et al. determined that miR-4417 was significantly down-regulated in keloid tissue and KFs, and increased miR-4417 expression led to the suppression of KFs proliferation and whereas miR-4417 depletion exerted an opposite effect, inferring the implication of miR-4417/CyclinD1 axis in keloid [81]. The overexpression of miR-1-3p/miR-214-5p could suppress the proliferation, migration, and invasion of human KFs, and promote apoptosis through AKT/ERK signaling pathway [82]. Mechanically, miR-1-3p or miR-214-5p was generally believed to bind to the 3′UTR of the TM4SF1, thus suppressing the expression of TM4SF1 [82].

**Fig. 1:**
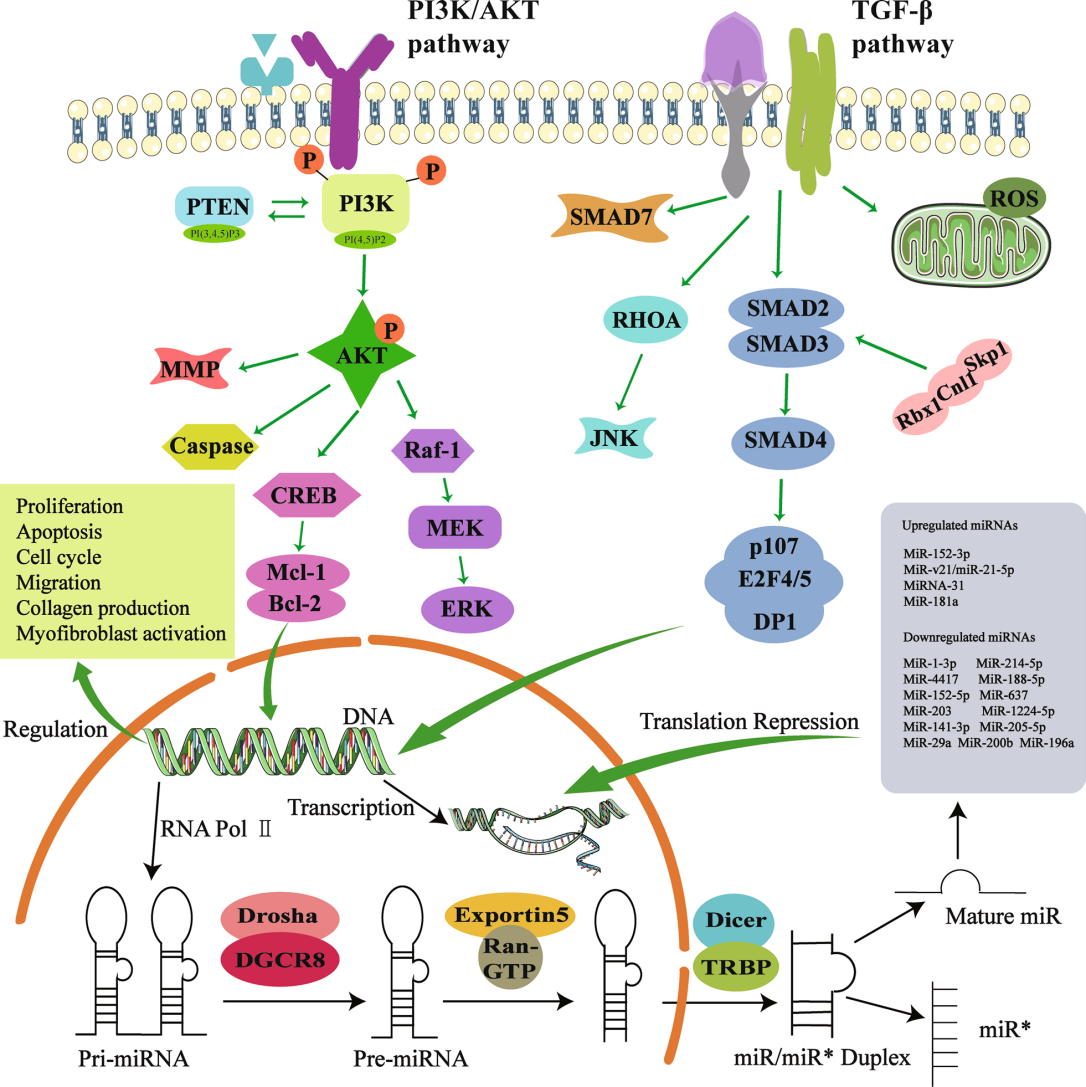
Schematic diagram of miRNA biogenesis and regulatory signaling pathways. MiRNAs are endogenous single-stranded RNAs with lengths of approximately 18–22 nucleotides. In the synthesis process of miRNAs, various enzymes are involved, including RNA polymerase, Drosha and Dicer enzymes, and Exportin-5 protein (a transporter protein). The pivotal role of mature miRNAs in regulating signaling pathways through binding to the 3′ untranslated region of the target mRNA, including PI3K/AKT signaling pathway and TGF-β signaling pathway. GTP: guanosine triphosphate; miRNA: microRNA; TRBP: TAR RNA binding protein

In aggregate, miRNAs are a highly heterogeneous group of ncRNAs in keloid, possessing keloid-specific expressions and distinct functions. All this evidence strongly demonstrates that in keloid, as the most reported ncRNA, miRNAs mainly function as the key regulators in the fibroproliferative process by inducing degradation of the corresponding mRNA or inhibiting post-transcriptional translation, thereby playing a key regulatory role in the molecular network of keloid pathogenesis (Fig. 1). These results open a new page for exploring the roles of miRNAs served as a novel diagnostic and therapeutic target of keloid (Table 4).

**Table 4:** Mechanisms and clinical value of ncRNAs in keloid

ncRNAs	Expression	Target gene	Mechanism	Clinical value	Ref
miR-152-3p	Upregulation	FOXF1	MiR-152-3p regulated cell proliferation, invasion and ECM expression through targeting FOXF1 in KFs	A novel and promising molecular target for keloid treatment	[63]
miRNA-31	Upregulation	HIF1AN	MiRNA-31 regulated proliferation, apoptosis, and cell cycle of keloid-derived fibroblasts by mediating HIF1AN/VEGF signaling pathway	A promising therapeutic target in keloid scarring	[64]
miR-181a	Upregulation	PHLPP2	MiR-181a targeted PHLPP2 to augment AKT signaling and regulate proliferation and apoptosis in human KFs	A therapeutic target for treatment of keloids	[65]
miR-21	Upregulation	TGF-β1	TGF-β1 increased the promoting actions of miR-21 on the proliferation and migration of KFs while attenuating apoptosis	A novel evidence on a theoretical basis for keloid treatment	[66]
miR-21	Upregulation	TGF-β1	TGF-β1 promoted KFs proliferation and transdifferentiation via up-regulation of miR-21 and PTENAKT signaling pathway	A potential theoretical basis for clinical treatment of keloids	[67]
miR-21-5p	Upregulation	PTEN	MiR-21-5p increased the migration, invasion, sphere-forming abilities of keloid keratinocytes, the phenotype of EMT, and cells stemness	A novel therapeutic targets for keloids	[68]
miR-21	Upregulation	FasL	MiR-21 regulated the apoptosis of KFs by caspase-8 and mitochondrial-mediated apoptotic signaling pathway	A therapeutic target for keloids	[69]
miR-21	Upregulation	Smad7	MiR-21 promoted Col1A1 and Col3A1 expression in keloid-derived fibroblast	A potential target for keloid treatment	[70]
miR-196a	Downregulation	COL1A1 and COL3A1	MiR-196a downregulation increased the collagens expression in KFs	A new therapeutic target for keloid lesions	[71]
miR-200b	Downregulation	N/A	MiR-200b inhibited the cell proliferation and promoted apoptosis of fibroblast via TGF-β1/a-SMA signaling	A useful target for hypertrophic scarring management	[72]
miR-29a	Downregulation	COL3A1	MiR-29a markedly reduced Type I and type III collagen mRNA and protein levels	A novel marker for keloids	[73]
miR-205-5p	Downregulation	VEGF	MiR-205-5p overexpression induced the cell apoptosis, and inhibited the cell invasion and migration ability in KFs	A potential therapy for prevention and treatment of keloids	[74]
miR-141-3p	Downregulation	GAB1	MiR-141-3p inhibited fibroblast proliferation and migration in keloids	A useful target for keloid management	[75]
miR-188-5p	Downregulation	N/A	MiR-1224-5p regulated proliferation, apoptosis, and invasion via the TGF-β1/Smad3 signaling pathway in KFs	A possible new therapeutic strategy for keloids	[76]
miR-203	Downregulation	EGR1 and FGF2	MiR-203 overexpression resulted in a significant decrease in proliferation, invasion, and ECM production in KFs	A potential role in preventing and treating keloids	[77]
miR-152-5p	Downregulation	Smad3	MiR-152-5p inhibited proliferation and migration and promotes apoptosis via the Erk1/2 and Akt pathways in human KFs	A potential therapeutic target of keloids	[78]
miR‑637	Downregulation	Smad3	MiR-637 inhibited KFs proliferation and metastasis	A promising therapeutic target in keloids	[79]
miR-188-5p	Downregulation	N/A	MiR-188-5p regulated proliferation and invasion via PI3K/Akt/MMP-2/9 signaling in keloids	A potential prognostic marker and therapeutic target for keloids	[80]
miR-4417	Downregulation	CyclinD1	MiR-4417 suppressed keloid fibrosis growth by inhibiting CyclinD1	A potential therapeutic target in keloids	[81]
miR-1-3p and miR-214-5p	Downregulation	TM4SF1	MiR-1-3p and miR-214-5p inhibited cell proliferation, migration, and induced apoptosis in HKFs	A potential targets in therapies for keloids	[82]
lncRNA-H19	Upregulation	miR-29a	LncRNA-H19 affected the viability and apoptosis of KFs through COL1A1 signaling	LncRNA-H19 was expected to allow for development of keloid-targeted treatments	[102]
lncRNAHOXA11-AS	Upregulation	miR-124-3p	High expression of HOXA11-AS essentially inhibited cell apoptosis and promoted fibroblast-induced angiogenesis via PI3K/Akt signaling pathway	A novel target for keloid therapy	[84]
lncRNA CAS1	Upregulation	N/A	LncRNA-CAS1 promoted calcium channel protein and type I collagen expression, and had a positive effect on cell migration in human KFs	A new therapeutic target for keloids	[85]
lncRNA-ATB	Upregulation	miR-200c	Knockdown of lncRNA-ATB decreased autocrine secretion of TGF-β2	Potential biomarkers and targets for novel diagnostic and therapeutic approaches for keloids	[86]
circRNA_0008259	Downregulation	N/A	Overexpression of circRNA_0008259 inhibited type I and collagen expression	Act as biomarkers of keloid	[47]
circCOL3A1-859267	Downregulation	miR-29c	circCOL3A1-859267 regulated type I collagen expression in fibroblasts	NA	[87]

The ncRNAs (miRNAs, lncRNAs, and circRNAs) are crucial in the coordination of KFs function and gene transcription, as well as in the pathogenesis and the prognostic value of keloid. *ncRNAs* non-coding RNAs, *miRNAs* microRNAs, *lncRNAs* long non-coding RNAs, *circRNAs* circular RNAs

### Mechanisms of lncRNAs in keloid

The lncRNA H19 appears to possess functional diversity and tissue specificity within distinct neoplasms and fibrosis. In the study by Wang et al., the lncRNA H19 and miR-29 were authenticated in the collected keloid, normal fibrous normal skin tissue [83]. The in vitro assay showed that lncRNA H19 might facilitate proliferation and metastasis of fibroblasts by modifying downstream miR-29a and COL1A1, engaging in the development of keloid-targeted treatments [83]. Based on lncRNA microarray and qPCR, lncRNA HOXA11-AS was finally identified as involved in the Wnt signaling pathway in keloid [43]. In addition, the expression levels of HOXA11-AS and TGFβR1 were observably up-regulated, whereas miR-124-3p was down-regulated in keloid tissue. Mechanistically, miR-124-3p was identified as a downstream effector involved in HOXA11-AS-mediated phenotypes through directly targeting TGFβR1, thus modulating the PI3K/Akt signaling pathway [84]. These findings revealed that through the miR-124-3p/TGFβR1 axis, HOXA11-AS inhibited cell apoptosis and promoted fibroblast-induced angiogenesis, contributing to the progression of keloid formation.

In a previous study, the expression of lncRNA CACNA1G-AS1 (CAS1) was confirmed abnormally high in keloid tissue, suggesting that CAS1 was involved in keloid formation [85]. Furthermore, Li et al. verified that CAS1 appeared to promote calcium channel protein and type I collagen expression, possessing a positive effect on cell migration in human KFs [85]. Using immunohistochemistry and qRT-PCR analysis, Zhu et al. showed that lncRNA-ATB and ZNF217, a transcriptional activator of TGF-β, were overexpressed while ZNF217-targeted miR-200c was under-expressed in keloid tissues and KFs [86]. The results also indicated that lncRNA-ATB governed the autocrine secretion of TGF-β2 in KFs by partially downregulating the ZNF217 expression via miR-200c, posing a signaling axis consisting of lncRNA-ATB/miR- 200c/ZNF217/TGF-β2 in keloid [86].

These above findings provided substantial evidence that the main lncRNA-driven mechanisms of keloid formation include regulation of differentiation and proliferation, cell cycle, apoptosis, ECM, and induction of Wnt signaling pathways. Nevertheless, lacking further verification of animal models of keloid in experimental design reduces the integrity of the above conclusions. Therefore, future investigations with more sophisticated designs and systematic argumentation are required to explore the effects of dysregulating lncRNAs in vivo keloid models and preclinical trials (Table 4).

### Mechanisms of circRNAs in keloid

The expression and abundance of ECM components are altered in KFs, especially the level of collagen. Based on the previous sequencing results, Zhang et al. further reported that overexpression of circRNA-0008259 using a lentiviral expression vector, significantly decreased the protein levels of type I and III collagen in KFs, proving that circRNAs were key factors to coordinate collagen hyperplasia and fibroblast proliferation [47]. They speculated that has-circ-0008259/miR-21-5p might regulate invasion, migration, sphere-forming, proliferation, and collagen deposition in KFs [47]. In another interesting study, Peng et al. showed that the transfection of a small interfering RNA targeting circCOL3A1-859267 or miR-29c mimic could obviously suppress the type I collagen expression in human dermal fibroblasts, also emphasizing the role of circCOL3A1-859267 in regulating type I collagen expression by sponging and sequestering miR-29c in human dermal fibroblasts [87]. Although emerging evidence has determined that the indispensable role of circRNAs in regulating the collagen deposition of KFs, this is only the tip of the iceberg. Currently, only a few research have investigated expression profiles of circRNAs in keloid, as well as the correlations and mechanisms of circRNAs in keloid. It is interesting to elucidate how circRNAs affect the biogenesis of keloid and regulate the various molecular mechanisms in KFs. The molecular networks connected with circRNA-miRNA-mRNA deserves further elaboration. In terms of clinical application prospects, the newly explored circRNAs with high sequence conservation, stability, tissue-specific expression, might be clarified as promising diagnostic biomarkers and therapeutic targets in keloid (Table 4).

## Conclusions and future perspectives

**Fig. 2:**
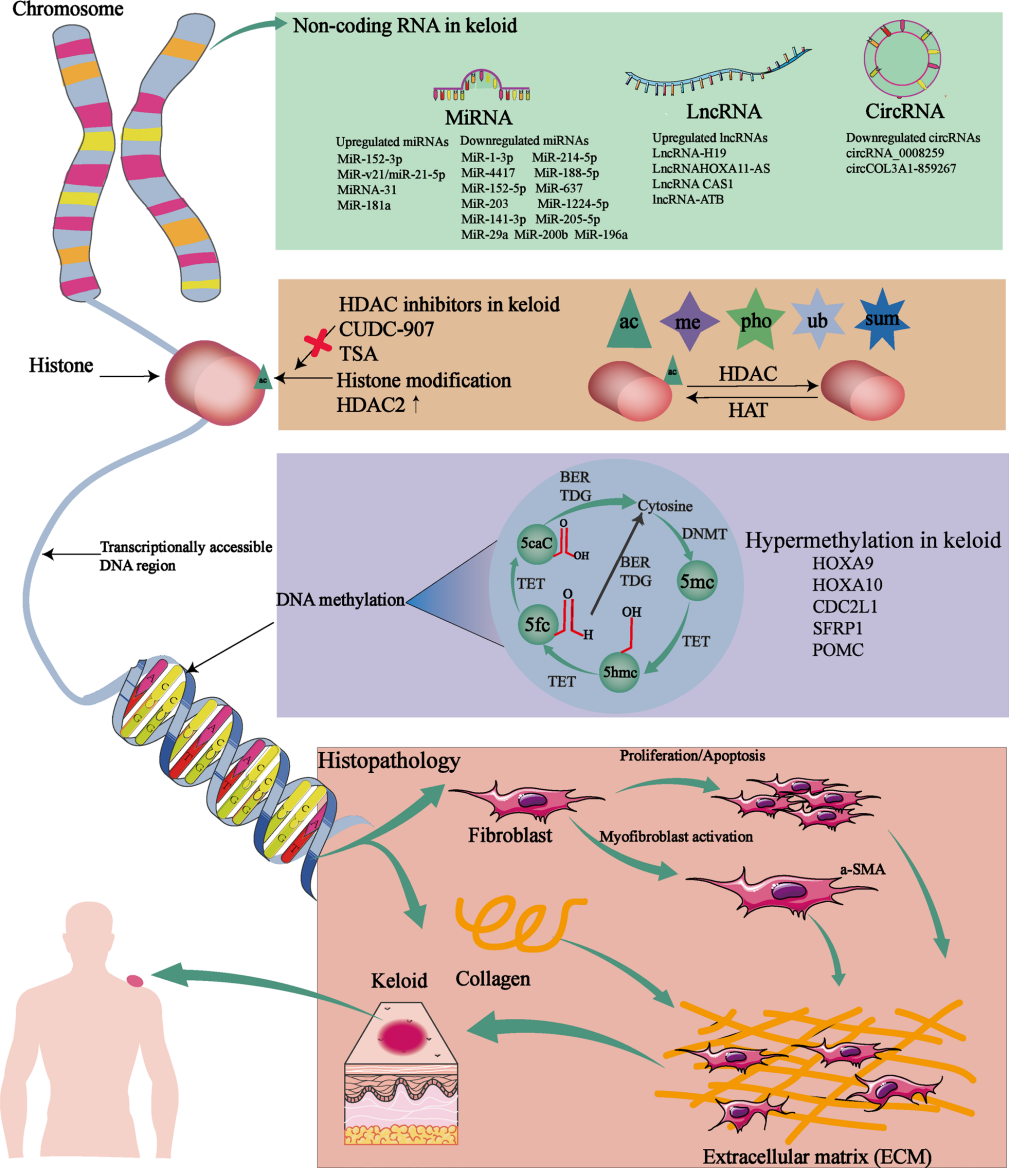
Illustration of the main epigenetic mechanisms (DNA methylation, histone modifications, and ncRNAs) involved in the regulation of keloid progression including aberrant proliferation, myofibroblast activation, apoptosis, cell cycle, migration, and collagen production. DNMT: DNA methyltransferase; *TET* ten-eleven translocation, *5mC* 5-methylated cytosine, *5fC* 5-formylcytosine cytosine, *5-hmC* 5-hydroxymethylated cytosine, *5caC* 5-carboxylcytosine cytosine, *TDG* thymine DNA glycosylase, *me* methylation, *ac* acetylation, *pho* phosphorylation, *ub* ubiquitination, *sum* sumoylation

The accumulating evidence has confirmed the epigenetic modification plays an indispensable role in keloid formation. Extensive gene silencing at the epigenetic level has been acknowledged as a rather important mechanism underlying keloid formation. Although epigenetic inheritance might not be passed on to the next generation, the numbers of the epigenetic alterations are retained until adulthood and correlated with gene expression, while the functional associations imply that keloid progress depends on the epigenetic modification. Therefore, as mentioned in this review, the roles and mechanisms of epigenetic modification in keloid are clarified, including DNA methylation, histone modification, and ncRNAs represented by miRNA, lncRNA, and circRNA (as illustrated in Fig. 2). Collectively, these findings reinforce the concept that there are huge, reciprocal, and complicated regulatory networks of epigenetics in keloid, emphasizing the potential application value of epigenetic markers in the keloid diagnosis, treatment, and prognosis, expectantly recognized as new candidates in the development of gene therapeutic strategies.

The genetic pattern in pedigrees with familial keloids revealed that keloid conformed to an autosomal dominant mode of inheritance with incomplete clinical penetrance and variable expression [88]. However, the occurrence of keloid does not seem to follow the simple Mendelian single-gene inheritance rule. On the contrary, keloids appear to be a polygenic disease, similar to those of nonpenetrance: other gene mutations or environmental factors might involve in the progression of the disease. The inheritance mode of keloid formation suggested that mutations in genes can predispose to keloid disorder. Through a multistage genome-wide association study in the Japanese population, Nakashima et al. determined four new susceptibility loci (rs873549, rs1511412, rs940187, and rs8032158) to keloids in three chromosomal regions (1q41, 3q22.3-23, and 15q21.3) [89]. The findings of Zhang et al. identified that a novel missense mutation in the CXCR1 gene existed in keloids and heightened CXCR1 expression was associated with keloid pathogenesis [90]. Interestingly, there is evidence suggesting that the pro allele of P53 Arg72Pro polymorphism increased genetic susceptibility for keloids in the Chinese population. However, there is no study in keloids to confirm the relationship between P53 gene mutation and genome stability. Although the study of the relationship between the epigenetic instability and keloid is barely reported, determining the causes and roles of genomic and epigenomic instability in keloid formation has the potential to yield more effective prevention strategies and therapeutics for keloid patients.

Nevertheless, there still are some challenges to the epigenetics modification in keloid. Firstly, the current studies mainly focus on the expression verification of clinical samples and in vitro cellular experiments, lacking enough in vivo animal studies. As keloid is unique to humans, adequate animal models will benefit the research aimed at prevention and effective therapeutic intervention. However, the existing animal models, including keloid scar implantation models and rabbit ear hyperplastic scar models, cannot totally, accurately reflect the disease characteristics of human keloid. The differences in human skin physiology and healing methods pose challenges to the keloid modeling of laboratory animals. Future animal models may utilize humanized mice with an immune system reconstructed using human immune cells, which might enable investigation of complex interactions between systemic and local factors that combine to promote keloid scar formation [91].

It is now explicit that mechanobiology forces are important in the degree of keloid formation, keloid contracture, and abnormal keloid progression. Additionally, the scientific basis and clinical evidence supporting the perspective that high mechanical loading resulting in ECM misalignment, proliferation and activation of fibroblasts, and, ultimately, excessive collagen production. For example, previous research by Dohi et al. has determined that keloid tends to occur in specific anatomical regions with high mechanical loading, including the anterior chest, shoulder, scapular, and suprapubic regions [92]. Through establishing a novel stretched scar model in rats, Zhou et al. found that scars could be generated by repeatedly and continuously applying mechanical forces on the edge of an incisional wound, and higher mechanical strain-induced significantly severer keloid progression [93]. Interestingly, we believe that epigenetics modification is involved in the mechanical regulation of keloid progression and functions. For example, Wang et al. found that wound edges treated by miR-21 antagomir have obvious wound contraction defects with impaired collagen deposition in the early stage of wound healing [94]. The study by Chen et al. also confirmed that the miR-21 could preserve the fibrotic mechanical memory in cells cultured on stiff substrates through regulating the acutely mechanosensitive myocardin-related transcription factor-A (MRTF-A/MLK-1) [95]. The above results provided direct evidence that miR-21 might be a mechanical sensitivity regulator in wound closure and the formation of keloids. However, in addition to miR-21, in terms of the direct relationship of epigenetic factors and mechanobiology factors in the keloid, there still seems to be a lack of enough evidence. It is still a research gap worthy of further investigation, which will help a comprehensive understanding of the process of wound healing, hypertrophic scar formation, and skin regeneration. Therefore, the mechanical force, epigenetics modification, and keloid formation, the relationship among the three is meaningful to be discussed in-depth in future research.

Lastly, the circRNAs are the emerging participators in keloid with unique functions in gene expression regulation. The high-throughput sequencing technologies have demonstrated that the circRNAs expression is observably different in keloid compared with normal skin tissue, suggesting that circRNA is partially responsible for shaping the clinical behavior of keloid. But, most of the existing research on circRNA in keloid is mostly focused on the expression level, but the intricate mechanisms and therapeutic potential of circRNA in the occurrence and development of keloid is yet to be fully substantiated.

As to date, the early diagnostic methods of keloid in clinical work, especially biochemical criterion, still are challenging tasks. For example, the application of fluorescence imaging technology with the molecular probes has some limitations in early keloid diagnosis, such as extremely shallow surface monitoring, cumbersome processes, and expensive costs [96]. It is worth noting that ncRNAs are pivotal participators in all the relevant phases of keloid like occurrence and progression, raising the diagnostic value of ncRNA in keloid by the broad liquid-biopsy category or by histopathological examination. Among different body fluid samples, serum proves to be the reliable samples in liquid biopsies. For example, the up-regulated miR-1225-5p, miR195-5p, miR-513-5p, and the down-regulated miR-6801-3p, miR-4254, and miRNA-412-3p were determined in the serum of patients with keloid through high-throughput sequencing and qRT-PCR verification, advocating that these six serum miRNAs are probable potential diagnostic or predictive biomarkers for further investigation in keloid [33]. Besides, a histopathologic detection found that circRNA-0008259 concentrations were degressive in patients of keloid accompanied by more aggressive collagen deposition in the keloid [47]. Based on characteristics such as highly conserved, stable, resistant to RNase R, and tissue-specific expression, circRNAs might have unparalleled charm and enormous potential in early diagnosis of keloid. Overall, it is of great value to excavate and build epigenetics-associated diagnostic strategies.

Even more interesting is the possibility of preventing disadvantageous epigenetic modifications for therapeutic purposes. The most relevant epigenetic modifications that play a critical role in keloid formation, are a source of potential therapeutic targets. The current experimental evidence regarding the role of epigenetic regulators including histone deacetylases proposes the therapeutic implication of these regulators in the keloid treatment. It also has confirmed that TSA, a classical HDAC inhibitor partly reverses TGF-β1 induced collagen synthesis and promotes apoptosis in KFs [97]. The result supports the epigenetically altered program in KFs, especially an altered pattern of histone acetylation, which provides novel and ponderable insights for keloid treatment. The epigenetic modification based on ncRNA also shows great application potential during keloid treatment. An exciting study found that EB irradiation inhibited autophagy in KFs by reducing miR-21-5p, which has already been confirmed to be closely involved in keloid progression, thereby indicating the significance of miR-21-5p-targeting therapy in controlling the keloid recurrence [98]. Furthermore, in a double-blinded, placebo-randomized, within-subject controlled clinical trial of single and multiple ascending doses of remlarsen in normal healthy volunteers, Gallant-Behm et al. found that miR-29b mimic (remlarsen) repressed ECM expression and the development of fibroplasia. These results suggest that miR-29b mimics might be an effective therapeutic to prevent the formation of keloid [99]. Since the complex interaction mechanism involving chromatin structure and ncRNA has not been fully elucidated, it is necessary to explore the value of combined therapy to produce synergistic and optimized effects, which is of great significance in future research, especially in clinical research [100].

Although a series of potential epigenetic markers for keloid diagnosis, prognosis, or response to treatment have been proposed, there are still many technical limitations in the process of translating into clinical practice. In terms of keloid diagnosis, it is well known that epigenetic responses are quickly and effectively adjusted based on environmental stimuli and the physiological state of the body. The main limitation in this instance to their application in clinical practice is the inconsistent repeatability and low accuracy of single index preclinical diagnosis. On the other hand, the main limitation of preclinical diagnosis is the difficulty in access to the keloid tissues in the body, and in most cases, preclinical epigenetic markers can only be detected after surgical resection. Furthermore, compared to tissue samples, blood samples are the easier access and storage but with a small proportion for keloid epigenetic diagnosis. Thus, there still a lack of early clinical diagnosis technology for keloid detection. The genetic difference of the patient, the degradability of ncRNAs, the influence of miscellaneous environment or internal factors, the lack of effective range and quality of biomarkers, and the time-consuming epigenetic testing with a high cost in routine clinical practice are also obstacles that need to be resolved before clinical application. Despite in its infancy, these epigenetic biomarker-based techniques are still of potential for keloid diagnosis, prognosis, or response to therapy that exist or are in development. Furthermore, signatures composed of a number of epigenetic methods and/or combined with genetic or biochemical detection methods will be necessary to improve the sensitivity and specificity of clinically useful biomarkers. For keloid treatment, the off-target effect caused by the lack of locus specificity is the Achilles heel of epigenetic drug therapy in the clinical application. For instance, as miRNAs regulate many genes or pathways at the same time to influence biological functions, the specific miRNA silence could result in unintended function changes of different targeted genes [101]. Additionally, the small sample size of the cohorts, the intrinsic heterogeneity of the diseases, the influence of confounding environmental or intrinsic factors are also barriers to be solved in the current epigenetic therapy. Besides, it is necessary to conduct clinical trials on a large scale and with long-term follow-up in order to ensure the efficacy and safety of epigenetic therapy.

In total, the epigenetics mechanisms represented by DNA methylation, histone modification, and ncRNA regulation are essential in keloid formation. In the subsequent studies, ongoing advances in epigenetics modification will unravel a deeper understanding of keloid etiology and offer novel and efficient diagnostic targets and interventions for keloid.

## Data Availability

Not applicable.

## Abbreviations

KFs: Keloid fibroblasts
TSA: Trichostatin A
GTP: Guanosine triphosphate
miRNA: MicroRNA
TRBP: TAR RNA binding protein. DNMT: DNA methyltransferase
TET: Ten-eleven translocation
5mC: 5-Methylated cytosine
5fC: 5-Formylcytosine cytosine
5-hmC: 5-Hydroxymethylated cytosine
5caC: 5-Carboxylcytosine cytosine
TDG: Thymine DNA glycosylase
me: Methylation
ac: Acetylation
pho: Phosphorylation
ub: Ubiquitination
sum: Sumoylation
N/A: Not available
DE: Differentially expressed
ncRNAs: Non-coding RNAs
miRNAs: MicroRNAs
lncRNAs: Long non-coding RNAs
circRNAs: Circular RNAs
qRT-PCR: Quantitative reverse transcription-polymerase chain reaction
KEGG: Kyoto Encyclopedia of Genes and Genomes
